# The Integration Paradox: A Phenomenological Study of Doula Services, Health Equity, and the Social Determinants of Perinatal Care

**DOI:** 10.3390/ijerph23050570

**Published:** 2026-04-28

**Authors:** Grace Mabiala-Maye, Keyonna M. King, Marisa S. Rosen, Regina Idoate, Michelle Strong, Chad Abresch

**Affiliations:** 1 Department of Health Services Research and Administration, University of Nebraska Medical Center (UNMC), Omaha, NE 68198, USA; 2 Department of Health Promotion, University of Nebraska Medical Center (UNMC), Omaha, NE 68198, USA; keyonna.king@unmc.edu (K.M.K.); marisa.rosen@unmc.edu (M.S.R.); regina.robbins@unmc.edu (R.I.); mistrong@unmc.edu (M.S.); cabresch@unmc.edu (C.A.)

**Keywords:** doula, maternal health equity, social determinants of health, Medicaid, perinatal care, health disparities, qualitative research, phenomenology

## Abstract

**Highlights:**

**Public health relevance—How does this work relate to a public health issue?**

- Maternal mortality in the United States reveals stark racial disparities, with Black women dying at rates three to four times higher than White women. Doula support has emerged as an evidence-based intervention to improve perinatal outcomes by addressing social determinants of health. Yet its integration into healthcare systems and community-based programs remains limited, particularly among populations experiencing the greatest disparities.

**Public health significance—Why is this work of significance to public health?**

- The central theme that emerges from experiences of doula integration is what we call the integration paradox, the tension between doula independence and the demands of healthcare delivery systems and care reimbursement structures, which arguably must be resolved for sustainable, equitable implementation. The findings show that cultural concordance between doulas and clients is essential for building trust, addressing social determinants of health (SDOH), and reducing disparities.

**Public health implications—What are the key implications or messages for practitioners, policy makers and/or researchers in public health?**

- The integration paradox operates across both healthcare delivery and reimbursement systems. To preserve the relationship-centered, advocacy-oriented approach that distinguishes effective doula care, Medicaid reimbursement policies and hospital policy reform is best undertaken in partnership with doula communities. The certification and credentialing debate represents a key dimension of the integration paradox: registry-based credentialing approaches may offer alternatives to formal licensure that document training without imposing barriers to community-based practitioners. Cultural concordance, identified as a facilitator of effective integration, should be prioritized in integration models to ensure doulas can effectively address SDOH for populations experiencing the greatest disparities.

**Abstract:**

The United States faces a maternal health crisis marked by stark racial disparities. Although doula support has emerged as an evidence-based intervention to improve perinatal outcomes by addressing social determinants of health, its integration into healthcare systems remains limited. This qualitative study, informed by phenomenological principles, examined multi-level experiences, perceived barriers, and perceived facilitators of integrating doula services into perinatal care systems and their intersection with health equity goals. We conducted 17 semi-structured interviews with 20 participants across Nebraska and Tennessee, including doulas, midwives, physicians, Medicaid administrators, and public health professionals, and analyzed data using reflexive thematic analysis guided by the Socio-Ecological Model. Three themes emerged: the integration paradox, an overarching theme capturing tensions between doula independence and healthcare system demands for standardization, including divergent views on practice models, provider dynamics, and certification; sustainable financing as the prevailing barrier, encompassing grant limitations, private pay inequities, absent Medicaid reimbursement, and the need for cost-effectiveness evidence; and cultural concordance as the prevailing facilitator, including cultural matching, addressing social determinants, and lived experience as motivation. Sustainable doula integration requires reconciling system demands for standardization with the relational, culturally responsive characteristics that define effective care, through Medicaid reimbursement pathways and policy reforms developed in partnership with doula communities.

## 1. Introduction

The United States faces a maternal health crisis despite high healthcare spending, with the highest maternal mortality rate among high-income countries and rates that have risen over the past two decades [1,2]. Black women had a mortality rate of 50.3 per 100,000 live births in 2023, nearly 3.5 times that of White women—a disparity that persists across education and income levels [3,4]. Structural racism significantly shapes these outcomes, with discrimination linked to 30% of pregnancy-related deaths in 2020 [5]. The cumulative physiological impact of chronic stress from racism, termed “weathering,” contributes to conditions that complicate pregnancy among Black women [6,7].

In this inequitable landscape, doula support has emerged as a promising, evidence-based intervention. While direct evidence linking doula care to reductions in maternal mortality remains limited, doula support addresses key risk factors for severe maternal morbidity, including cesarean delivery, preterm birth, and inadequate prenatal care, which are proximal contributors to pregnancy-related complications and death. A Cochrane systematic review found that continuous labor support—particularly from doulas—increases spontaneous vaginal birth rates, shortens labor, reduces use of pain medication, and lowers cesarean rates [8]. While the Cochrane review did not disaggregate outcomes by race, subsequent studies suggest that doula care benefits may be particularly pronounced among Black birthing people. Research using Medicaid claims data found that women receiving doula care had 52.9% lower odds of cesarean delivery and 57.5% lower odds of postpartum depression or anxiety than matched controls [9]. These benefits appear particularly pronounced among marginalized populations, with culturally concordant doula support showing promise for reducing racial disparities in birth outcomes [10].

Doulas practice across diverse settings, each with distinct characteristics relevant to integration efforts. Private practice doulas serve clients who can afford out-of-pocket fees, typically ranging from $800 to $2500 [11]. Hospital-based doulas are employed by healthcare systems and assigned to patients during labor, which may limit continuity of the relationship. Community-based doulas practice within reproductive justice frameworks, often serving populations facing health disparities through sustained relationships from prenatal care through postpartum [10]. Doula training and certification vary widely, with organizations such as DONA International, CAPPA, and community-based training programs each setting different requirements [12]. These distinctions—in practice setting, philosophical orientation, and credentialing pathways—are essential for understanding how different integration models may affect doula effectiveness and health equity outcomes.

For marginalized populations, community-based doulas provide benefits that extend beyond clinical outcomes. These doulas practice within a reproductive justice framework, guiding clients to resources that address social determinants of health, which remain less accessible to Black and Brown birthing people due to structural racism [10]. Research has demonstrated that racial congruence and shared lived experience foster trusting relationships that mitigate racism’s effects in maternity care [13,14].

Despite this evidence base, doula services remain inaccessible to many who would benefit most. Private doula care costs $800 to $2500, placing it beyond the reach of low-income families [11]. Hospital-based doula programs may reduce cost barriers but often lack the relationship continuity and cultural concordance that distinguish community-based models. Medicaid covers approximately 42% of US births but has historically not reimbursed doula services in most states, creating a profound equity barrier: those who could benefit most often lack access [12,15].

Recent years have seen growing policy interest in closing this access gap. By late 2024, over 20 states had implemented or were implementing Medicaid coverage for doula services [11,16]. The 2020 Surgeon General’s Call to Action and the 2022 White House Blueprint both recommended expanded coverage [17]. Yet implementation has been complex, as states navigate questions of credentialing, certification, and reimbursement structures [12].

The challenges of doula integration extend beyond financing. Questions about the scope of practice, relationships with clinical providers, and the fundamental orientation of doula care toward client advocacy rather than institutional compliance shape how doulas can be incorporated into existing healthcare structures [18]. Licensure and credentialing present particular complexities: unlike licensed healthcare professionals such as nurses or midwives, doulas currently operate without standardized licensure requirements in most states, and the question of whether and how to credential doulas remains contested among community- and healthcare system-based interest-holders [12]. These tensions are particularly salient for health equity: the very characteristics that may make doulas effective—their embeddedness in communities, independence from healthcare systems, and capacity to advocate for clients—may conflict with the standardization and accountability demands that healthcare systems and payers require [11,12]. Recent research documents distinct challenges facing private practice, hospital-based, and community-based doulas, including financial barriers, institutional resistance, and sustainability concerns [19].

The Socio-Ecological Model (SEM) provides a theoretical framework for understanding how health behaviors and outcomes are shaped by factors operating at multiple levels: individual, interpersonal, organizational, community, and policy [20]. In the context of doula integration, individual-level factors include doula training, motivation, and lived experience; interpersonal factors encompass doula–client relationships and doula–provider dynamics; organizational factors involve healthcare system policies and employment structures; community factors address cultural concordance and community-based program capacity; and policy factors include Medicaid reimbursement and credentialing requirements. This multi-level framework is particularly suited to examining doula integration, as interactions across these levels shape the phenomenon—for example, policy decisions about certification requirements affect community-based doulas’ ability to maintain the interpersonal relationships that drive their effectiveness.

This qualitative study examines multi-level perspectives on integrating doulas into perinatal care systems in Nebraska and Tennessee, with particular attention to how structural barriers and facilitators intersect with health equity goals. Nebraska, a Medicaid expansion state, has structured hospital-based maternity care, an active perinatal quality improvement collaborative, and community doula programs serving populations facing health disparities. However, Medicaid reimbursement for doula services has not yet been established. The healthcare landscape includes academic medical centers, community hospitals, federally qualified health centers, and independent midwifery practices. Tennessee, a non-expansion state, offers insight into community-driven doula models. Nashville hosts various maternal health programs, including federally qualified health centers with midwifery services, public health initiatives such as Nashville Strong Babies, community doula groups, and participation in multi-site collaborative projects focused on integrating doula services into healthcare settings. While individual elements of the tensions surrounding doula integration, including questions of independence versus accountability, financing barriers, and cultural concordance, have been documented in prior research [19,21,22,23], no study has named and analytically defined these tensions as a coherent paradox or examined how they operate across multiple levels of the Socio-Ecological Model through the perspectives of diverse stakeholders in contrasting policy contexts. This study contributes by articulating the integration paradox as an analytical framework, revealing how policy-level decisions about credentialing and financing interact with community-level and interpersonal-level factors that drive doula effectiveness, and identifying conditions under which the paradox may be more or less pronounced. By examining perspectives across multiple levels of influence, we sought to illuminate the dynamics shaping doula integration and identify pathways toward sustainable, equitable models of care that address the social determinants of perinatal health.

## 2. Materials and Methods

### 2.1. Study Design and Theoretical Framework

This qualitative study, informed by phenomenological principles and analyzed using reflexive thematic analysis, examined multi-level experiences of doula integration, focusing on how participants perceive and make sense of the challenges and facilitators of integrating doula services within perinatal care systems. A phenomenological sensibility guided the study’s orientation toward participants’ lived experiences and meaning-making, while reflexive thematic analysis provided the systematic analytical procedure for generating themes across a diverse, multi-stakeholder sample [24,25]. This approach was chosen because it highlights participants’ lived experiences and interpretations, revealing structural and interpersonal factors that influence doula integration as perceived by those involved [26,27]. By engaging doulas, healthcare providers, administrators, and policymakers, the study identified both shared and distinct perspectives on challenges and opportunities.

Using a qualitative approach guided by the Socio-Ecological Model [20], we gathered perspectives from doulas, midwives, physicians, Medicaid administrators, perinatal quality collaborative leaders, and public health professionals across Nebraska and Tennessee. The SEM guided participant selection to ensure representation across multiple levels of influence: individual (doulas, midwives, physicians), organizational (healthcare systems, community health centers, doula programs), and policy (Medicaid administrators, public health officials, quality improvement leaders). The SEM also informed data analysis by sensitizing the research team to how factors at each level interact to shape the phenomenon of doula integration. The study followed COREQ guidelines for transparent reporting of methods, covering team characteristics, design, data collection, analysis, and findings [28]. The complete checklist is provided in the Supplementary Materials.

### 2.2. Research Team and Reflexivity

The lead researcher, a female physician with a Master of Public Health specializing in Maternal and Child Health and currently pursuing doctoral studies, conducted all interviews. Her background includes clinical maternal health experience, research on qualitative methods, health equity, and perinatal care systems, and community engagement in maternal health initiatives. No one else was present during interviews except the participant(s) and the interviewer. The six-member research team included faculty supervisors with expertise in qualitative research, maternal and child health policy, health equity, and health services. The senior author oversaw data collection and analysis and independently coded some transcripts to ensure rigor.

No prior relationships existed before recruitment. Initial contact was made through outreach to networks and referrals. During consent, the lead researcher disclosed her background, including medical training, dedication to maternal health equity, credibility, rapport, and to acknowledge her positionality [29]. Participants were told the study aimed to explore their experiences of doula integration—defined for participants as the processes through which doula services are incorporated into healthcare systems and community-based programs—with insights intended to inform strategies for improved doula integration. The lead researcher’s roles as a clinician and health services researcher enabled her to engage with both clinical and policy aspects. Still, she had to remain reflexive about potential biases stemming from her background.

The lead researcher maintained a reflexivity journal throughout data collection and analysis to examine how her positionality, assumptions, and experiences might influence the research process [26]. Journal entries documented emotional responses, interpretations, and methodological decisions, creating an audit trail. Discussions with the senior author helped to identify potential biases, particularly those related to her focus on health equity and maternal care systems.

### 2.3. Study Setting Selection

This study was conducted in two US regions: the Midwest (Omaha, Nebraska) and the Southeast (Nashville, Tennessee). These locations were purposively selected to capture diverse healthcare landscapes, policy environments, and approaches to doula integration, enabling examination of structural factors that influence integration across contexts [30].

### 2.4. Participant Selection

#### 2.4.1. Sampling Strategy

Participants were recruited through purposive and snowball sampling to ensure diverse representation across roles, organizations, and perspectives on doula integration [31]. The SEM guided purposive sampling to ensure representation across individual practice (doulas, midwives, physicians), organizational contexts (healthcare systems, community health centers, doula programs), and policy levels (Medicaid administrators, public health officials, quality improvement leaders). Eligibility required participants to have at least two years of experience in doula services, maternity care, or maternal health policy, ensuring they could provide meaningful insights. Snowball sampling was used, with initial participants identifying others, prioritizing racial and cultural diversity to reflect perspectives on culturally concordant doula care [32].

#### 2.4.2. Recruitment

Potential participants were identified through outreach to doula programs, health agencies, healthcare systems, and networks. The team contacted organizations that work with doulas and support maternal health in both regions and requested referrals. Referred community- and healthcare system-based interest-holders were emailed a recruitment letter describing the study purpose, procedures, and voluntary nature of participation. No incentives were offered. The high participation rate (91%) likely reflects the salience of this topic among professionals actively engaged in maternal health work.

### 2.5. Data Collection

#### 2.5.1. Interview Format and Procedures

Data were collected through semi-structured interviews, with the format chosen based on participant preferences and logistical constraints. Most interviews were conducted individually (*n* = 15). Two multi-participant sessions were conducted: one triad interview with three Medicaid specialists and one paired interview with two Nashville health professionals. These multi-participant sessions were chosen when participants requested a joint format or when scheduling necessitated this approach; they were not designed as traditional focus groups. The triad comprised three Medicaid reimbursement specialists from the same state agency, and the paired interview included two public health professionals working on community-based doula integration in Nashville. The same semi-structured guide was used across all sessions. Group dynamics may have encouraged consensus-oriented responses in multi-participant sessions, though they also elicited perspectives that emerged through professional dialog and collegial exchange. During analysis, the research team attended to interactive dynamics, noting where participants converged, diverged, or modified their positions in response to others. Interviews were conducted via Zoom or in person at participants’ workplaces, based on preference and availability. All interviews were audio-recorded with consent. Only the participant(s) and lead researcher were present to ensure confidentiality and minimize external influence.

#### 2.5.2. Interview Guide

A semi-structured interview guide was developed to explore participants’ experiences with doula models, challenges and facilitators of integration, and sustainability strategies (see Table A1 in Appendix A). The guide was informed by the SEM, with questions organized to progress from individual-level experiences (e.g., doula training, motivations, and practice) through interpersonal and organizational dynamics (e.g., doula-provider relationships, healthcare system structures) to community and policy contexts (e.g., cultural concordance, Medicaid reimbursement, credentialing). It also leveraged the literature on doula services, health workforce integration, and maternal health equity. Questions were open-ended to allow participants to share experiences and address key topics [33]. The interview addressed the following overarching questions: What are participants’ experiences of doula integration? What are the perceived challenges of integration? What are the perceived facilitators of integration? How can these insights inform strategies for effective implementation? Interview items were organized to address these overarching questions across the multiple SEM levels described above, ensuring that each question was explored from individual, organizational, and policy perspectives. The interview guide covered participants’ backgrounds, views on doula models, barriers at various levels, relationships with care providers, financing, training, and sustainability. Questions explored experiences during prenatal, labor, and postpartum phases. The interview guide was pilot-tested with two eligible individuals not included in the final sample. Minor revisions improved the clarity and flow of the question. During interviews, the guide was used flexibly, with adjustments to question order and emphasis to follow participants’ narratives while covering key topics.

#### 2.5.3. Field Notes

The lead researcher took field notes in two stages [34]. During interviews, she recorded brief handwritten notes on contextual observations, including participant affect, nonverbal cues, and environmental setting. Immediately following each session, these in-session notes were expanded into more detailed reflective memos capturing initial impressions, emerging analytical ideas, and observations about interview dynamics. These memos were used during analysis to contextualize transcript interpretation.

#### 2.5.4. Data Saturation

Thematic saturation was assessed iteratively throughout data collection [35]. The research team reviewed transcripts and field notes after each session, monitoring the emergence of new codes and themes. By the fifteenth individual interview, no new themes were emerging, and the final two multi-participant sessions served to elaborate and confirm existing patterns rather than introduce new constructs. Saturation was assessed at the level of overarching themes across the full sample rather than within individual stakeholder strata, which is a limitation of the study design. The concept of “information power” [36], also supports the adequacy of the sample: the specificity of the research aim, the theoretical framework guiding data collection, the inclusion of highly experienced participants, and the quality of dialog across interviews contributed to sufficient analytical depth [35].

### 2.6. Data Analysis

#### 2.6.1. Transcription and Data Management

All interviews were transcribed verbatim by a professional service. The lead researcher reviewed each for accuracy and re-immersion. Transcripts were de-identified, replacing participant names with identifiers and redacting identifying details. Transcripts and data were managed with the Dedoose software (Version 9.0; SocioCultural Research Consultants, LLC, Los Angeles, CA, USA).

#### 2.6.2. Analytical Approach

Data were analyzed using reflexive thematic analysis [24,25], selected for its flexibility and researcher involvement. The process comprised six iterative phases: familiarization, coding, developing themes, reviewing themes, defining/naming, and reporting. These phases were not strictly sequential, but involved ongoing movement as understanding deepened.

#### 2.6.3. Coding Process

The lead researcher began by open-coding early transcripts to generate initial codes grounded in participants’ language and experiences. The coding framework was developed iteratively, guided by research objectives and the Socio-Ecological Model as a sensitizing framework, while remaining open to new insights. Codes were systematically applied across all transcripts, and the codebook was refined over time to incorporate new insights and merge overlapping codes.

To enhance analytical rigor, the senior author independently coded approximately 20% of transcripts (four transcripts, representing a cross-section of participant roles and study sites, reflecting multiple levels across the SEM). The two coders met regularly to compare decisions, discuss discrepancies, and refine definitions. Through collaborative engagement and dialog, the two coders expanded the analytical framework to support richer interpretations. The purpose of dual coding was to deepen the analysis through multiple interpretive perspectives rather than to achieve statistical agreement, consistent with the reflexive, researcher-led nature of thematic analysis [25].

#### 2.6.4. Theme Development

Themes were developed inductively from coded data, noting meaning patterns and perspective differences linked to roles, context, or location. Initial themes were checked against the whole dataset to ensure representation and identify challenging data. They were refined iteratively, with broad constructs becoming specific subthemes in a hierarchy.

### 2.7. Trustworthiness

Multiple strategies were used to establish trustworthiness, covering credibility, transferability, dependability, and confirmability [37,38]. Credibility was enhanced by drawing on multiple data sources and perspectives to enrich interpretation, including cross-referencing interview transcripts with field notes and comparing perspectives across roles and locations [39]. Participant feedback on preliminary interpretations was solicited by sharing emerging themes with a subset of participants to assess resonance and accuracy. Participants verified that the themes reflected their experiences, and minor clarifications refined the final themes. Although full transcripts were not returned, member checking of interpretive findings allowed participant validation. Transferability was supported by detailed descriptions of the research context, participant characteristics, and analytical procedures, enabling readers to assess applicability to other settings [40]. Including two distinct geographic and policy contexts increases potential relevance across diverse settings, though local factors may influence specific insights. An audit trail documented all research steps, including recruitment, interviews, coding, and theme development, ensuring transparency and dependability. Reflexive practices, such as journaling and team discussions, helped to confirm the findings by examining researcher biases. Quotations extensively support interpretations using participants’ words.

### 2.8. Ethical Considerations

This study was reviewed by the University of Nebraska Medical Center IRB and determined not to be human subjects research under 45 CFR 46.102, as it involved interviews with professionals about their experiences rather than research involving personal health data or vulnerable groups. Still, the team followed ethical principles for qualitative human research.

All participants provided written informed consent before participating, confirming their understanding of the study’s purpose, procedures, risks, and benefits. The consent forms stated that participation was voluntary, that participants could withdraw at any time without penalty, and that responses would be de-identified in reports and publications. Participants also consented to audio recording and were informed about data storage and confidentiality protections.

Confidentiality was maintained by restricting access to identifiable data to the research team and storing all recordings and transcripts on encrypted, password-protected systems with limited authorization. The study was not fully anonymous, as participants were known to the interviewer; however, de-identification procedures were used to protect participant identity in all outputs. These procedures included replacing names with numeric identifiers, redacting or generalizing identifying details in transcripts and reports, and attributing quotes by professional role only. Findings are presented in aggregate to prevent identification.

## 3. Results

Data were collected from January to March 2024 through 15 individual interviews, one triad interview, and one paired interview, for a total of 17 sessions with 20 participants. Interviews ranged in duration from 30 to 90 min. Of the 22 individuals approached, 20 participated (91%); two declined due to scheduling conflicts. The triad interview included three Medicaid specialists who shared insights on reimbursement and policy, fostering dialog on agency consensus and differences. The paired interview included two Nashville health professionals who discussed community-driven integration initiatives.

The final sample reflected multiple levels of influence according to the Socio-Ecological Model, including perspectives from individual practice (doulas, midwives, physicians), organizational contexts (healthcare systems, community health centers, doula programs), and policy levels (Medicaid administrators, public health officials, quality improvement leaders). The sample comprised four doulas (one private practice, one hospital-based, and two community-based), six maternal and child health specialists, three health system researchers, three Medicaid reimbursement specialists, two physicians, two midwifery/nursing professionals, and four community-based organization representatives. Because some participants held multiple roles, these expertise categories are not mutually exclusive (Table 1). Organizational type and size were intentionally varied: community-based programs (*n* = 4), maternal and child health organizations *(n* = 3), universities (*n* = 4), hospitals (*n* = 4), and state agencies *(n* = 5). Organizations served maternity populations of varying sizes: small (<1000 births; *n* = 4), medium (1000–3000; *n* = 7), and large (>3000; *n* = 9). Saturation was reached after 17 interviews, with no new themes emerging and existing themes well elaborated across perspectives. Participant details are presented in Table 1.

Three themes emerged from the analysis: (1) the integration paradox, the overarching theme encompassing tensions between doula independence and healthcare system requirements, including the certification and credentialing debate; (2) sustainable financing challenges, the prevailing barrier; and (3) cultural concordance, the prevailing facilitator. Within each theme, participants articulated distinct perspectives shaped by their professional roles and organizational contexts. Notably, perspectives varied not only between the two states but also within each state, reflecting the diversity of roles and organizational contexts represented in each setting. Table 2 summarizes the three themes, their associated subthemes, key findings, and representative quotes from participants.

The three themes operate across multiple levels of the Socio-Ecological Model. Theme 1, the integration paradox, primarily reflects tensions at the organizational and policy levels but is grounded in individual-level experiences of doula practice; this theme includes the certification and credentialing debate, which operates at the intersection of policy-level requirements and community-level access to the profession. Theme 2, sustainable financing, operates predominantly at the policy level (Medicaid reimbursement, legislation) with consequences at the organizational level (program sustainability, hospital employment structures). Theme 3, cultural concordance, functions as a facilitator across multiple SEM levels: it encompasses individual-level factors (lived experience, motivation), interpersonal dynamics (doula–client trust, cultural matching), and community-level resources (community-based training programs, reproductive justice organizations).

### 3.1. Theme 1: The Integration Paradox (Overarching Theme)

The integration paradox emerged as the overarching theme characterizing experiences of doula integration among both community- and healthcare system-based interest-holders. A fundamental tension pervaded participants’ discussions of doula integration: the characteristics that make doulas effective—their independence from healthcare institutions, their relationship-based approach, and their advocacy orientation—are precisely the features that complicate integration into existing healthcare structures. This theme emerged across all sectors interviewed, though with varying emphases and proposed resolutions.

#### 3.1.1. Subtheme 1.1: Doula Independence and Client Advocacy

Doulas consistently articulated their role as fundamentally oriented toward client advocacy rather than institutional compliance. As one experienced doula explained:

*“We work for clients, not the healthcare system. That’s the whole point. If you’re employed by the hospital, who are you really advocating for when there’s a conflict?”*

(Doula)

This perspective was shared across doula participants, who expressed concern that hospital employment would compromise their ability to support clients whose preferences might conflict with institutional protocols or provider recommendations. Another doula described the relationship-building that occurs outside clinical settings:

*“I go to baby showers. I’m there when they’re cooking dinner, when they’re worried about something at two in the morning. That relationship is what makes me effective when we get to the hospital. If I just met someone in active labor, how could they trust me?”*

(Doula)

Several doulas recounted experiences in which their advocacy role put them in tension with clinical staff. One participant described witnessing a consent violation:

*“The resident was doing a vaginal exam, and my client said, ‘Stop, this hurts, please stop. And they didn’t stop. They kept going. That’s the kind of thing I’m there to witness and to help my client process afterward. If I worked for that hospital, could I really speak up about that?”*

(Doula)

#### 3.1.2. Subtheme 1.2: Healthcare System Accountability and Standardization

Healthcare administrators and clinical leaders acknowledged the value doulas provide while emphasizing the need for clear accountability structures. A Medicaid administrator explained the constraints facing state programs:

*“We can’t pay for services rendered by just anybody. There has to be some standard by which we can enroll them. Licensure or certification—we need something. As much as we’d like to support doulas, we can’t add to sustainability through payment unless that happens.”*

(Medicaid Specialist)

A physician participant framed the issue in terms of care coordination, emphasizing the importance of communication loops within healthcare teams:

*“The connections between people caring for a patient have to be tight—not just touching, but overlapping. The doula has to be willing to provide information about the patient to the clinician. Otherwise, it becomes a missing link, and we don’t want weak links.”*

(Physician)

The administrator of a perinatal quality improvement collaborative offered a nuanced perspective, acknowledging both the value of doula independence and the need for system integration:

*“Doulas should be an integral part of the healthcare team—not the sole solution, but a critical piece for reducing disparities. But they also want to be welcomed, acknowledged, included in teaching moments. That means we need doula-friendly policies, and those have to be developed with community doula input, not imposed on them.”*

(Quality Improvement Administrator)

#### 3.1.3. Subtheme 1.3: Community-Based Versus Hospital-Based Models

The debate over community-based versus hospital-employed doula models crystallized the integration paradox. Public health professionals and doulas generally favored community-based approaches, while some healthcare administrators saw hospital employment as more feasible for integration and payment. A national maternal–child health organization director articulated the community perspective:

*“It’s easy to build it out of a hospital or health system. But is that the right way? I would lean toward no—if it’s built out of the hospital, it’s probably too medical model and not going to be as supportive as it could be for birthing people. Community-based doulas understand the food, the traditions, what a family looks like. That’s what supports people through pregnancy.”*

(Public Health Professional)

A neonatologist who directs a perinatal quality collaborative offered a practical perspective on the tradeoffs:

*“It really depends on the structure of different systems and what the payment model is. If hospital systems have close partnerships with prenatal clinics and can incorporate doula services throughout pregnancy, that’s one reasonable way. But I’ve heard that when there’s no continuity—when you get a different doula from a pool for your visits and delivery—it doesn’t work as well. Women want to form a bond, have someone who knows their whole pregnancy.”*

(Physician)

The CEO of a national maternal–child health organization captured the consensus view among those prioritizing health equity:

*“Doulas should exist in prenatal care, labor and delivery, pediatric care, postpartum care—and outside clinical settings. Home visits, community spaces, over coffee. Going to diminish the value of doulas if they’re part of the hospital’s administrative and staffing system. They should not be part of that.”*

(Public Health Professional)

#### 3.1.4. Subtheme 1.4: Provider Relationships and Professional Dynamics

Participants described varying relationships between doulas and different provider types. A certified nurse-midwife who had previously practiced as a doula offered insight into these dynamics:

*“It depends on the doula. The majority of the time, it’s relatively positive. There are doulas I sing hallelujah when I see them in the birth room—they’re calm, logical, reassuring. But occasionally, advocacy can progress to the point that it interferes with the provider-patient relationship. It’s one thing to advocate; it’s another to interfere with medical care.”*

(Midwife)

An experienced doula who is also a registered nurse described the professional dynamics differently:

*“Obstetricians are pathology experts, surgeons, responsible for the life. Midwives are experts on normal and physiologic birth. Doulas are also experts on physiologic normal birth. That’s why midwives and doulas work better together—we share a philosophy. The doctor is always looking for where the problem is going to be. We’re trying to keep people healthy and low-risk so they don’t develop the pathology that needs to be managed.”*

(Doula)

To address relationship challenges, some institutions have developed innovative approaches. One midwife described a bridge-building initiative:

*“We started something called Birth Talk, where once a quarter we invite local doulas to have a potluck with providers. We talk about why treating gestational diabetes is important, what happens if there’s meconium, what about the heart rate do we care about. We do role play. We see each other as people and professionals to improve understanding of where the other person is coming from.”*

(Midwife)

#### 3.1.5. Subtheme 1.5: The Certification and Credentialing Debate

The question of certification requirements crystallized the integration paradox, revealing tensions between healthcare system demands for standardized credentialing and doula community concerns about maintaining access to the profession. A national MCH organization director noted:

*“The minute you license something, you make it out of reach for some people. Doula care and birth workers have existed since the beginning of time. I’m less a fan of licensing and more a fan of figuring out some common understanding of what a doula is and what skill sets are. Then we figure out reimbursement.”*

(Public Health Professional)

A doula expressed similar concerns:

*“I understand the need for standards, but I’m worried about barriers to entry. Different certifying bodies have different requirements—mine was 22 h training, plus exams, plus annual fees. Licensing could create obstacles to equitable access to becoming a doula. I support a registry concept—doulas register, document training and experience—without onerous requirements.”*

(Doula)

The perinatal quality collaborative administrator offered a middle-ground perspective:

*“Healthcare systems want standards and oversight; doulas are not medical professionals and shouldn’t be restricted like licensed providers. But there could be a registry system where doulas list training and experience. Various state approaches exist. When the MCO asked us for a white paper, we recommended DHHS work directly with doulas rather than speaking for them.”*

(Quality Improvement Administrator)

This subtheme exemplifies the integration paradox: the very mechanisms designed to facilitate integration through standardization risk undermining the community-based access that makes doulas effective for populations experiencing the greatest disparities.

### 3.2. Theme 2: Sustainable Financing

Sustainable financing emerged as the most consistently identified barrier to doula integration across all participants. Participants described a landscape characterized by temporary grant funding, private pay arrangements inaccessible to low-income families, and the absence of sustainable reimbursement mechanisms through Medicaid or private insurance.

#### 3.2.1. Subtheme 2.1: Grant Funding Limitations

Multiple participants described reliance on grant funding as inherently unsustainable. A public health professional articulated the challenge:

*“Medicaid reimbursement is critical for doula employment. Grants can provide short-term or pilot funding, but without Medicaid reimbursement, the long-term prospects of using doulas are very limited. We’ve seen it so many times—the grant ends, the program goes away. Maybe it’s resurrected later, but we have to think long-term.”*

(Public Health Professional)

A doula program coordinator described the practical impact of funding instability:

*“Right now, we’re sustainable because we have grant funding. But when the grant runs out, we need insurance to help us pay for it. We know one doula won’t be enough—we’re delivering over 700 babies a year. To truly reach the patients we need to reach, we need more.”*

(Public Health Professional)

The quality improvement collaborative administrator noted the strategic purpose of grant funding:

*“The point of grant funding isn’t to fund doulas temporarily—it’s funding to create opportunity for communities to figure out a long-term solution. We’re watching pilot programs in other states closely, but they’re all doing it differently, which is where we’re at in the genesis of developing a reimbursement model.”*

(Public Health Professional)

#### 3.2.2. Subtheme 2.2: Private Pay and Equity Barriers

Participants described private pay arrangements as creating stark equity barriers. Doula fees in the study regions ranged from $600 to $2500, depending on experience and services included. While hospital-based doula programs absorb costs within institutional budgets, eliminating direct charges to patients, community-based doula programs typically rely on grant funding to subsidize services for low-income clients, and private practice doulas charge fees that reflect market rates. A midwife observed:

*“I’ve had people want doulas but be unable to afford them. The cost has gone up exponentially—when I started, a doula cost $150 to $200. Now I’ve seen doulas charging $2500. It’s not that they’re not valuable, but those costs are outside the reach of many people.”*

(Midwife)

A doula described her fee structure and the challenges of serving low-income clients:

*“I charge $1275 total, split into three payments. I’ve worked with grant-funded programs, but when full grant funding covers everything without client investment, sometimes people don’t engage the same way. But the women who need doulas most can’t afford what I charge.”*

(Doula)

A Nashville public health professional articulated the equity implications directly:

*“If you’re pregnant, you should be able to get a list of doulas that insurance will reimburse and interview them. If doulas only want to stay independent and only serve people who can pay $2000 out of pocket, then what about the women who can’t pay? Their access to doulas is cut off simply because they don’t have disposable income.”*

(Public Health Professional)

#### 3.2.3. Subtheme 2.3: Medicaid Reimbursement Barriers

State Medicaid administrators explained the structural barriers to reimbursement. A DHHS administrator noted:

*“The doula has not been assigned a clinical reimbursement position within Medicaid. To get reimbursed, you have to have a contract with the payer, you have to be contractually aligned to provide services. The codes exist—the same prenatal, labor, and delivery codes. But the doula may not have those contracts.”*

(Health Policy Expert)

Another Medicaid specialist elaborated on the certification requirements:

*“States need to be thoughtful about certification or licensure requirements. You want to set it up so individuals can achieve certification—you don’t want it to be a barrier to becoming a provider type. But you also want enough requirements that the individual becomes a respected member of the care team.”*

(Medicaid Specialist)

A Medicaid administrator offered a framework for what legislation would need to include:

*“Any bill should require the Department of Public Health to implement some certification, give an expectation that Medicaid covers doulas, and come with an appropriation of funding. We would work with local and national experts to define what services would be and estimate costs.”*

(Medicaid Specialist)

Participants noted that other states have made progress while Nebraska has lagged. A doula training program leader observed:

*“We testified to the Nebraska Senate Health and Human Services Committee advocating for Medicaid reimbursement. But there’s been pushback. White-led organizations opposed to the bill language, and white women doulas don’t see as many Black women as Black doulas do. They don’t see the struggles.”*

(Doula)

#### 3.2.4. Subtheme 2.4: Value Proposition and Return on Investment

Multiple participants emphasized that demonstrating cost-effectiveness would be essential for advancing policy change. An obstetrician-administrator articulated this perspective:

*“Can we demonstrate that hiring a doula has a return on investment? We know outcomes are better with labor support, but we don’t know that doulas perform better than family members. It would be an added cost to the healthcare delivery system—therefore important to demonstrate it’s cost-effective.”*

(Physician)

A neonatologist noted the type of evidence legislators require:

*“Working with our legislature, state-level data can be incredibly helpful. They want to see quantitative data—they’re not going to want to hear that women felt more supported. They want to see we saved NICU days, we saved cesarean sections. It’s simple math for them.”*

(Physician)

A community health center administrator framed the argument for policymakers:

*“If doulas working with midwives and providers save moms’ lives and babies’ lives, they’re also helping for healthy pregnancies and healthy births. That reduces moms in critical care, babies in the NICU. We’re not talking hundreds of thousands of dollars for doulas—we’re talking reasonable wages for hard work that saves millions on the healthcare system.”*

(Public Health Professional)

### 3.3. Theme 3: Cultural Concordance (Prevailing Facilitator)

Participants consistently identified cultural concordance—the matching of doulas with clients who share racial, ethnic, or cultural backgrounds—as the prevailing facilitator of effective doula integration. This theme connects to broader discussions of how doulas address the social determinants of health and why community-based models may be more effective for marginalized populations.

#### 3.3.1. Subtheme 3.1: Cultural Matching and Trust

A public health director articulated the importance of culturally matched doula support:

*“Having community-based doulas, doulas who understand all the nuances of the culture—the food, the traditions, what a family looks like—supports your birthing person in ways not normally received if you’re just going for prenatal visits. Doulas can help mitigate stress, and they can advocate. We know medical systems don’t often listen to Black and brown birthing people.”*

(Public Health Professional)

A reproductive justice program associate described her organization’s training program:

*“Our Doula Passage Program is 14-week training full-spectrum doulas. We bring in cultural competency for Black maternal families, nutrition, meetings with midwives. We’re training Black women from the community to serve Black mothers. The hospital-based doula model—you meet this stranger in active labor—there’s no relationship, no trust. How can you build trust in the middle of active labor?”*

(Doula)

A neonatologist who has partnered with community organizations endorsed this approach:

*“I would love to see culturally matched doula support for all of our minority women or birthing people. Having someone who can represent and understand you from a cultural standpoint is going to be more supportive than someone who doesn’t have that shared experience.”*

(Physician)

#### 3.3.2. Subtheme 3.2: Addressing Social Determinants of Health

Participants described how doulas address social determinants that extend beyond clinical care. A public health professional explained:

*“When you provide support to a birthing person, that encompasses all that’s around that person—the social determinants of health, their physical and emotional well-being. If they’re stressed because of bills, if there’s domestic violence—doulas are in the middle of that. And preparing traditional foods postpartum, that fourth trimester support that Western society has gotten away from—there’s no reimbursement for that.”*

(Public Health Professional)

A doula described the breadth of support she provides:

*“My role can support any family transition—birth, postpartum, even abortion, death, elderly care, child welfare reunification. I intentionally partner with organizations serving families of color, single mothers, young mothers without support. Doula work is natural village and community support that American society has removed.”*

(Doula)

An obstetrician acknowledged the value of this expanded role:

*“Perhaps a greater role for doulas is as a health navigator throughout pregnancy—helping people navigate the healthcare system, particularly important for people who are not native Americans and may not understand the US healthcare system. Currently, doulas are underutilized in that setting because they tend to be limited to labor only.”*

(Physician)

#### 3.3.3. Subtheme 3.3: Lived Experience as Professional Motivation

Several doulas described how personal experiences of inequitable care motivated their professional commitment. A doula program leader shared:

*“My endometriosis was misdiagnosed as ‘crotch lightning.’ I required emergency surgery a year postpartum—lost an ovary and tubes. That’s why I became a doula. I know what it’s like to not be heard, to have your pain dismissed. This work is a calling.”*

(Doula)

Another doula described her path into the work:

*“The birth of my oldest son—I studied, read, planned how I wanted my birth to be. And my birth was nothing like that. Everything I did not want, except for having a C-section. How could I have this much information and still not get what I wanted? That fire was lit. Before doula was even a profession, your grandmothers, aunties, sisters—those were the original doulas.”*

(Doula)

**Figure 1:**
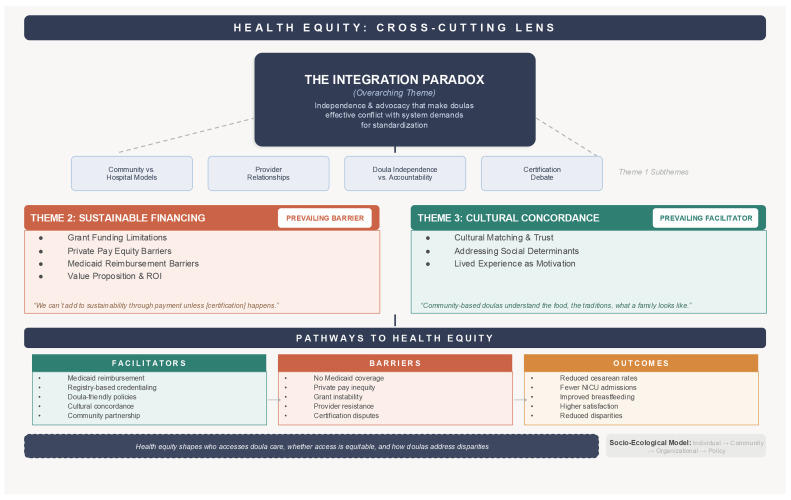
Conceptual framework for doula integration and health equity.

Figure 1 illustrates the conceptual framework emerging from this analysis, depicting the integration paradox as the overarching theme shaped by two interconnected themes—sustainable financing (prevailing barrier) and cultural concordance (prevailing facilitator)—along with the facilitators, barriers, and outcomes that define pathways to health equity. Health equity is represented as a cross-cutting lens that frames all three themes, reflecting its role as an integrating concept rather than a standalone theme.

The integration paradox—wherein the independence and advocacy that make doulas effective conflict with healthcare system demands for standardization—is illuminated by Theme 2 (sustainable financing challenges that perpetuate the paradox) and Theme 3 (cultural concordance as a facilitator that explains why doula independence matters for addressing perinatal disparities). The certification and credentialing debate, positioned as a dimension of the integration paradox (Theme 1), illustrates how quality-assurance mechanisms can either facilitate or impede equitable integration, depending on their design. The framework illustrates facilitators, barriers, and potential outcomes along pathways to health equity, informed by participant perspectives across multiple levels of the Socio-Ecological Model. This figure is intended as a visual synthesis of the thematic relationships identified in the analysis rather than a generalizable theoretical model; further research across additional contexts would be needed to test its broader applicability.

## 4. Discussion

This qualitative study examined multi-level experiences of doula integration across healthcare, policy, and community contexts. Three themes emerged: the integration paradox as the overarching theme—including the certification and credentialing debate as a key dimension of this paradox—with sustainable financing as the prevailing barrier and cultural concordance as the prevailing facilitator. Health equity, rather than constituting a separate theme, emerged as a cross-cutting lens through which all three themes intersect: the integration paradox matters because it shapes who can access doula care; sustainable financing determines whether access is equitable; and cultural concordance is the mechanism through which doulas most effectively address disparities. This study reveals a fundamental paradox at the heart of doula integration efforts: the characteristics that make doulas effective—independence from healthcare institutions, relationship-based care, and client advocacy—are precisely the features that complicate integration into existing healthcare structures. Our findings contribute to a growing body of literature examining structural and policy barriers to doula integration [21,22,23], while extending this work to illuminate how health system demands conflict with the community-based, culturally responsive characteristics that distinguish doula care. Analytically, the integration paradox emerges when three conditions converge: (a) the intervention derives its effectiveness from characteristics that are non-clinical and relationship-based; (b) the delivery system requires standardization, credentialing, and accountability structures for reimbursement and quality assurance; and (c) the target population experiences structural disadvantages that make community embeddedness and cultural concordance essential to effectiveness. This framework connects to the Consolidated Framework for Implementation Research, which emphasizes the tension between an intervention’s core components and the inner setting demands of healthcare organizations; our findings suggest that resolving the paradox may require adapting the setting rather than the intervention.

### 4.1. Reconciling Independence and Integration

Our findings suggest that the binary framing of community-based versus hospital-employed models obscures more nuanced possibilities. Participants across all sectors interviewed expressed a preference for financial integration (Medicaid reimbursement, insurance coverage) while resisting structural integration that would compromise doula autonomy. This distinction is critical: sustainable financing does not require hospital employment, and insurance reimbursement can be structured to preserve the community-based, relationship-centered approach that participants identified as essential. These findings align with Kozhimannil and colleagues’ (2016) observation that doula care operates according to a fundamentally different logic than medicalized birth, emphasizing continuous support and client empowerment rather than clinical intervention [23].

The tension between doula independence and healthcare system accountability reflects broader debates about the professionalization of community health workers [41]. The effectiveness of doulas may derive in part from their position outside the medical hierarchy, allowing them to advocate for client preferences even when those conflict with institutional protocols [42]. The certification and credentialing debate, which emerged as a key dimension of the integration paradox (Theme 1), illuminates these tensions: healthcare systems require credentialing mechanisms for quality assurance, yet overly restrictive requirements could exclude community members who bring cultural knowledge and lived experience—the very attributes that may make doulas most effective for populations experiencing health disparities [43]. Registry-based approaches may offer a middle path that documents training and experience without imposing barriers associated with formal licensure, aligning with flexible models identified by the National Academy for State Health Policy [44].

### 4.2. Sustainable Financing as a Health Equity Issue

The financing landscape creates stark equity barriers. When doula services cost $1500 to $2500 and are not covered by insurance, access becomes determined by ability to pay—precisely inverting the distribution that health equity goals would dictate. This pattern reflects what Crear-Perry and colleagues (2021) have termed “structural racism in maternal health,” wherein populations at greatest risk face the most significant barriers to accessing evidence-based interventions [45]. Grant funding, while valuable for pilot programs, does not provide sustainable financing for scaled implementation.

The cost-effectiveness argument deserves attention from policymakers and researchers. While evidence for the impact of doula care on clinical outcomes is well established [8], participants noted that state legislators often require state-specific data demonstrating cost savings. Kozhimannil and colleagues (2013) estimated that Medicaid reimbursement could yield net savings of $58.4 million annually through reduced cesarean deliveries alone, but translating national estimates to state-level projections requires local data that may not be available in states without Medicaid doula coverage [46].

Our inclusion of Nebraska and Tennessee provided perspective on how different policy contexts shape doula integration experiences. Tennessee, despite not having expanded Medicaid, has developed community-based doula programs through the Nashville Strong Babies initiative and participates in collaborative projects focused on doula integration. In contrast, Nebraska, which has expanded Medicaid, has not established mechanisms for doula reimbursement. The contrasting experiences of participants in these two states raise questions about the relative contributions of Medicaid expansion, political will, and local advocacy to doula integration, though the sample composition (16 Omaha, 4 Nashville) does not allow systematic comparison [30].

### 4.3. Cultural Concordance and Social Determinants

Participants’ emphasis on cultural concordance connects doula care to broader frameworks of social determinants of health. Doulas who share cultural backgrounds with clients can address dimensions of perinatal care that extend beyond clinical encounters: food traditions, family structures, spiritual practices, and the cumulative impact of discrimination within healthcare systems. This expanded understanding challenges models that narrowly focus on labor support and suggests the need for reimbursement structures that recognize the full scope of doula services across pregnancy and postpartum.

Our findings resonate with Hardeman and Kozhimannil’s (2016) [43] examination of the motivations of doulas of color entering the profession. That personal experiences of being unheard or mistreated within healthcare systems motivated professional commitment to doula work suggests an important dynamic: community-based programs may draw practitioners whose personal histories equip them to support clients navigating similar challenges.

This aligns with promotora and community health worker models, in which shared life experiences enhance professional effectiveness [47]; however, it raises questions about workforce sustainability. If doulas are motivated primarily by mission rather than compensation, the field may struggle to retain practitioners at the scale necessary to impact population-level disparities.

### 4.4. Implications for Policy and Practice

Our findings suggest several considerations for policymakers and healthcare systems, consistent with recommendations from the March of Dimes and the ACOG [48,49]. First, Medicaid reimbursement policies should be developed in partnership with doula communities, ensuring that participation requirements do not inadvertently exclude community-based practitioners or constrain the relationship-centered approach that distinguishes doula care. Requirements perceived as burdensome or exclusionary may undermine the equity goals that doula coverage is intended to advance.

Second, hospital policy reforms should be developed with input from community doulas rather than imposing top-down requirements. The perinatal quality collaborative model offers a promising approach wherein hospital policies are developed through collaborative processes, including doula representation.

Third, investments in doula workforce development should prioritize cultural concordance, supporting training programs that recruit from and are accountable to communities experiencing the greatest disparities. Research suggests that racial concordance between patients and providers is associated with improved communication, trust, and outcomes, particularly for Black patients [50,51].

Fourth, evaluation efforts should generate evidence that policymakers require while remaining sensitive to the complexity of doula care. The mechanisms through which doulas improve outcomes—relationship building, advocacy, social support—may not be fully captured by clinical outcome measures; mixed-methods approaches combining outcome evaluation with process and implementation research may be necessary.

### 4.5. Theoretical Implications

Our findings deepen understanding of how non-clinical workforce models relate to health equity. The Socio-Ecological Model helps to identify perspectives across levels, but the integration paradox shows that interventions at different ecological levels can conflict. Specifically, our themes map onto SEM levels in instructive ways. For example, the integration paradox (Theme 1) reflects tensions primarily at the organizational and policy levels, where healthcare system demands for standardization clash with doula practice models rooted in individual and interpersonal dynamics; the certification debate, as a component of this theme, operates at the intersection of policy-level credentialing requirements and community-level access to the profession. Sustainable financing challenges (Theme 2) operate predominantly at the policy level, yet their consequences cascade downward, affecting organizational program sustainability and individual doula livelihoods. Cultural concordance (Theme 3) functions as a facilitator that emerges from individual-level lived experience and interpersonal trust. Yet, its effectiveness depends on community-level resources and policy-level decisions about credentialing that determine who can practice as a doula. This cross-level dynamic means that well-intentioned policy changes to increase doula access might, if designed without community input, inadvertently limit the factors that make doulas effective.

This has implications for implementation science. The Consolidated Framework for Implementation Research emphasizes “fit” between interventions and settings. Our findings suggest that doula care may require adapting healthcare and policy systems to integrate it, rather than changing doulas themselves. Policymakers should consider how systems can accommodate doula care while maintaining its core features.

### 4.6. Limitations

This study has several limitations. First, the sample of 20 participants across two geographic regions, while appropriate for qualitative inquiry focused on depth of understanding, limits the transferability of specific findings. The themes identified should be understood as reflecting stakeholder perceptions within these particular settings rather than as universally applicable conclusions. The findings may not fully transfer to states with established Medicaid doula coverage or to states with different healthcare market structures. However, the conceptual themes—tensions between independence and accountability, financing as an equity issue, and the importance of cultural concordance—likely have broader relevance.

Second, the perspectives of birthing people who have used or been unable to access doula services were not directly captured. This was a deliberate methodological decision: as Patton (2015) notes, service providers and service recipients occupy fundamentally different experiential positions, and assessing both using the same instrument and analytical approach within a single study risks conflating distinct phenomena [30]. A separate study designed to center birthing people’s experiences, with its own interview guide, sampling strategy, and interpretive framework, would be needed to capture the recipient perspective on doula integration. Future research should prioritize this complementary line of inquiry, particularly among communities experiencing the greatest perinatal disparities.

Third, the study focused on individual perspectives rather than direct observation of doula care or measurement of outcomes. While qualitative approaches informed by phenomenological principles are appropriate for understanding lived experience and professional perspectives [27], they cannot assess the effectiveness of different integration models. Mixed-methods research combining qualitative inquiry with quantitative outcome evaluation would strengthen the evidence base.

Fourth, the sample included only one obstetrician, limiting our understanding of physician perspectives. Direct engagement with a larger sample of obstetricians would provide a more comprehensive understanding of clinical provider perspectives on doula integration.

### 4.7. Future Research Directions

Further research areas include comparing community-based, hospital-employed, and hybrid models to guide Medicaid reimbursement policies. Studying states as they implement Medicaid doula coverage can reveal factors that help or hinder success. Long-term research on doula workforce growth, retention, and career development can help to build a sustainable, diverse workforce. Participatory research involving doulas and birthing people as co-investigators will ensure that findings are relevant to community needs.

## 5. Conclusions

Integrating doula services into perinatal care offers opportunities and challenges for health equity. Our findings show the integration paradox as the overarching theme: a tension between doulas’ relationship-based, advocacy approach and the standardization, accountability, and credentialing needed by healthcare systems and payers. The certification and credentialing debate, a key dimension of this paradox, requires approaches—such as registry-based models—that document training without creating barriers to community-based practitioners. Resolving this paradox requires sustainable financing, especially via Medicaid reimbursement, while maintaining doula care’s unique qualities.

Cultural concordance is key to addressing the social determinants that shape perinatal disparities, and community doula programs hold promise for addressing social factors that affect perinatal outcomes among marginalized groups. The US faces a maternal health crisis with persistent racial and socioeconomic disparities resistant to clinical efforts [4]. Doula care provides not only clinical support but also addresses structural vulnerability, discrimination, and social marginalization, influencing perinatal health.

As states and healthcare systems consider doula integration, the findings from this study suggest that policies may benefit from partnering with doula communities, recognizing the full scope of doula services beyond labor support, and preserving the community-based, relationship-centered nature of doula care. The broader literature on doula effectiveness, combined with the perspectives of participants in this study, suggests that developing models that scale access while maintaining the core principles that make doulas valuable remains a central challenge. Only by navigating the integration paradox—achieving sustainability without sacrificing effectiveness—can the promise of doula care for health equity be fulfilled.

## Figures and Tables

**Table 1:** Participants’ Characteristics.

Characteristic		*N*	%
Participant expertise	Doulas (1 private, 1 hospital-based, 2 community-based)	4	20 *
	Maternal and Child Health Specialist	6	30 *
	Health System Researcher	3	15
	Medicaid Reimbursement Specialist	3	15
	Physicians	2	10
	Midwifery/Nursing	2	10 *
Organization type	Community-Based Organization	4	20
	Maternal and Child Health Organization	3	15
	University	4	20
	Hospital	4	20
	State-level organization	5	25
Regional location	Nashville	4	20
	Omaha	16	80
Size of maternity populations served	Small (<1000 births per year)	4	20
	Medium (1000–3000 births per year)	7	35
	Large (>3000 births per year)	9	45

* Several participants held additional secondary roles in these categories that are not reflected in the primary counts; categories were assigned based on each participant’s primary professional role.

**Table 2:** Summary of themes on doula integration, sustainability, and health equity.

Theme	Subtheme	Key Findings	Representative Quote
Theme 1: The Integration Paradox	Doula Independence and Advocacy	Doula participants described their role as fundamentally oriented toward client advocacy rather than institutional compliance. Hospital employment was perceived as potentially compromising the ability to advocate when client preferences conflict with institutional protocols.	“We work for clients, not the healthcare system. That’s the whole point. If you’re employed by the hospital, who are you really advocating for when there’s a conflict?” (Doula)
	Healthcare System Accountability	Administrators emphasized the need for clear accountability structures, certification standards, and communication loops within healthcare teams. Participants noted that Medicaid requires enrollment standards for reimbursement.	“We can’t pay for services rendered by just anybody. There has to be some standard by which we can enroll them. Licensure or certification—we need something.” (Medicaid Specialist)
	Community-Based vs. Hospital Models	Doulas and public health professionals expressed preference for community-based models. Hospital employment was described as “too medical model.” Relationship continuity from prenatal through postpartum is identified as essential.	“If it’s built out of the hospital, it’s probably too medical model and not going to be as supportive. Community-based doulas understand the food, the traditions, what a family looks like.” (Public Health Professional)
	Provider Relationships	Participants described doula–midwife relationships as generally positive due to a shared philosophy of physiologic birth. Doula–physician relationships were reported as more variable. Bridge-building initiatives (e.g., “Birth Talk”) help build mutual understanding.	“Midwives are experts on normal physiologic birth. Doulas are also experts on physiologic normal birth. That’s why midwives and doulas work better together—we share a philosophy.” (Doula/RN)
	Certification Debate	Participants identified tension between quality assurance needs and barriers to entry. Some noted that licensure could exclude community members with cultural knowledge. Registry-based approaches were suggested as a middle path, documenting training without onerous requirements.	“The minute you license something, you make it out of reach for some people. Doula care has existed since the beginning of time. I’m less a fan of licensing and more a fan of figuring out common understanding.” (Public Health Professional)
Theme 2: Sustainable Financing	Grant Funding Limitations	Multiple participants characterized grant funding as temporary and unsustainable. Programs were reported to disappear when grants end. Participants noted that grant funding is valuable for pilots but insufficient for scaled implementation.	“We’ve seen it so many times—the grant ends, the program goes away. Maybe it’s resurrected later, but we have to think long-term.” (Public Health Professional)
	Private Pay Equity Barriers	Participants reported doula fees ranging from $600 to $2500, creating equity barriers. Several noted that women who would benefit most cannot afford services, and that access is determined by the ability to pay rather than need.	“If doulas only serve people who can pay $2000 out of pocket, then what about the women who can’t pay? Their access is cut off simply because they don’t have disposable income.” (Public Health Professional)
	Medicaid Reimbursement Barriers	Medicaid specialists explained that doulas lack a clinical reimbursement position within Medicaid. Enrollment requires contracts with payers and certification standards. Participants indicated that legislative action is needed to establish coverage and appropriate funding.	“The doula has not been assigned a clinical reimbursement position within Medicaid. To get reimbursed, you have to have a contract with the payer, be contractually aligned to provide services.” (Health Policy Expert)
	Value Proposition and ROI	Participants reported that legislators require quantitative, state-specific data demonstrating cost savings—evidence needed on reduced NICU stays, cesarean rates, and maternal morbidity. Cost-effectiveness analysis is essential for policy advancement.	“They want to see we saved NICU days, we saved cesarean sections. It’s simple math for them. State-level data can be incredibly helpful.” (Physician)
Theme 3: Cultural Concordance	Cultural Matching and Trust	Participants described culturally concordant doulas as building deeper trust through understanding of cultural nuances, food traditions, and family structures. Programs like Doula Passage train Black women from the community to serve Black mothers.	“Having someone who can represent and understand you from a cultural standpoint is going to be more supportive than someone who doesn’t have that shared experience.” (Physician)
	Addressing Social Determinants	Participants described doulas as addressing SDOH beyond clinical care, including stress from bills, domestic violence, food insecurity, and postpartum traditions. The doula role was seen as extending to health navigation and connecting families with community resources.	“When you provide support to a birthing person, that encompasses all that’s around that person—the social determinants of health, their physical and emotional well-being.” (Public Health Professional)
	Lived Experience as Motivation	Several doulas described personal experiences of inequitable care as motivating their professional commitment. Those who experienced dismissal, misdiagnosis, or birth trauma reported bringing empathy and determination to support others facing similar challenges.	“My birth was nothing like what I planned. Everything I did not want. How could I have this much information and still not get what I wanted? That fire was lit.” (Doula)

Note: Quotes are attributed by professional role to maintain confidentiality while providing context.

## Data Availability

The qualitative data generated during this study are not publicly available due to privacy and confidentiality concerns. The study participants did not consent to their interview data being shared publicly. However, de-identified analytical materials may be available from the corresponding author upon reasonable request, subject to privacy restrictions.

## Supplementary Materials

The following supporting information can be downloaded at: https://www.mdpi.com/article/10.3390/ijerph23050570/s1, Table S1: Consolidated Criteria for Reporting Qualitative Studies (COREQ): 32-item checklist.

## Abbreviations

The following abbreviations are used in this manuscript:

ACA	Affordable Care Act
COREQ	Consolidated Criteria for Reporting Qualitative Research
DHHS	Department of Health and Human Services
HHS	Health and Human Services (US Department of)
IRB	Institutional Review Board
MCH	Maternal and Child Health
MCO	Managed Care Organization
MMRC	Maternal Mortality Review Committee
NICU	Neonatal Intensive Care Unit
PMSS	Pregnancy Mortality Surveillance System
PRMR	Pregnancy-Related Mortality Ratio
RN	Registered Nurse
ROI	Return on Investment
SDOH	Social Determinants of Health
SEM	Socio-Ecological Model
UNMC	University of Nebraska Medical Center

## Appendix A

The semi-structured interview guide was developed to explore individual perspectives on doula integration, sustainability, and health equity. Questions were informed by the Socio-Ecological Model framework and addressed experiences across prenatal, labor, and delivery, and postpartum phases of care.

**Table A1:** Interview Guide.

Research Question and/or Interview Intent	Interview Questions	Possible Probes/Reminders for Me
Intro & warm-up	Can I start by asking you to just tell me a little bit about what you do and why it’s meaningful to you? How do you feel about a doula’s services? What do you think the role of a doula should be within the healthcare system? In your opinion, what are the key benefits of having doula services more readily available?	Establish rapport and get them comfortable. Explore perceptions of doulas in different settings (private, hospital-based, community-based). Ask for specific examples, particularly related to prenatal care, labor/delivery, and postpartum support.
What are the perceived challenges of integration?	From your experience or perspective, what are the main challenges in integrating doula services into the existing perinatal care systems? Can you provide specific examples or instances where you’ve observed these challenges?	Credentialing, funding, reimbursement, acceptance in clinical settings, and awareness among patients and providers Structural, financial, institutional, or policy-related barriers
What are the perceived facilitators of integration?	What do you think would help promote greater use of doula services? Can you tell me about a time when doula services were successfully integrated into a prenatal care clinic or hospital?	Training programs, standardization, partnerships, Medicaid/private insurance reimbursement. Probe for a story! Ask for real-world examples in hospitals, clinics, or community programs.
How can these insights inform policymakers for effective implementation?	What kind of policy changes or systemic reforms do you think are necessary to integrate doula services effectively? How can policymakers better understand and address the challenges in integrating doula services? Looking forward, what do you envision as the future of doula services within perinatal care systems? What steps do you believe are essential for the sustainable integration of doula services? Is there anything else you would like to share about your experiences or views on integrating doula services in perinatal care?	Legislative efforts, reimbursement policies, and public health initiatives Explore gaps in knowledge and engagement from policymakers Institutional support, funding mechanisms, stakeholder collaboration Encourage broad reflection and discussion. Allow participants to provide any final thoughts or insights.
